# AudBility: an online program for central auditory processing screening in school-aged children from 6 to 8 years old

**DOI:** 10.1590/2317-1782/20232022011

**Published:** 2023-08-28

**Authors:** Nádia Giulian de Carvalho, Maria Isabel Ramos do Amaral, Maria Francisca Colella-Santos

**Affiliations:** 1 Programa de Pós-graduação em Saúde, Interdisciplinaridade e Reabilitação, Departamento de Desenvolvimento Humano e Reabilitação - DDHR, Universidade Estadual de Campinas - UNICAMP - Campinas (SP), Brasil.; 2 Departamento de Desenvolvimento Humano e Reabilitação - DDHR, Faculdade de Ciências Médicas - FCM, Universidade Estadual de Campinas - UNICAMP - Campinas (SP), Brasil.; 3 Centro de Investigação em Pediatria, Faculdade de Ciências Médicas - FCM, Universidade Estadual de Campinas - UNICAMP - Campinas (SP), Brasil.

**Keywords:** Children, Students, Hearing Tests, Screening, Auditory Perception, Crianças, Estudantes, Testes Auditivos, Triagem, Percepção Auditiva

## Abstract

**Purpose:**

To analyze the performance of students aged between in an auditory skills screening software program, considering the influence of biological determinants and the correlation of auditory tasks with the behavioral assessment tests of central auditory processing (PAC), as well as to present the cutoff points of the battery.

**Methods:**

In the first stage, the sample consisted of 96 students with typical development, who underwent hearing screening at school. A self-perception questionnaire and the auditory tasks of sound localization (SL), temporal resolution (TR), temporal ordering of frequency (OT-F) and duration (OT-D), auditory closure (AC), dichotic digit- binaural integration (DD) and figure-ground (FG) were applied. Of these, 66 children participated in the second stage of the study, including basic and behavioral audiological assessment from PAC.

**Results:**

The gender variable influenced the DD task to the right ear. Age influenced the outcome of five auditory tasks. The right ear performed better in the DD and OT-F tasks. At the age between 6 and 7 years, there was a correlation between screening and diagnosis in the tasks of AC, TR, DD, FG, and OT-F. At the age of 8 years, there was a correlation in the DD and OT-F tasks. The pass/fail criteria varied according to the task and biological determinants.

**Conclusion:**

There was a correlation between screening and diagnosis in a greater number of tasks in the age group between 6 and 7 years. The cut-off points for the auditory tasks should be analyzed according to age, sex and/or ear side.

## RESUMO

**Objetivo:**

Analisar o desempenho de escolares em um programa de triagem das habilidades auditivas, considerando a influência de determinantes biológicos e a correlação das tarefas auditivas com os testes da avaliação comportamental do processamento auditivo central (PAC), bem como apresentar os pontos de corte da bateria.

**Método:**

Na primeira etapa a amostra foi constituída por 96 escolares com desenvolvimento típico, que passaram pela triagem auditiva escolar. Foi aplicado o questionário de autopercepção e as tarefas auditivas de localização sonora (LS), resolução temporal (RT), ordenação temporal de frequência (OT-F) e duração (OT-D), fechamento auditivo (FA), dicótico de dígitos- integração binaural (DD) e figura-fundo (FF). Dessas, 66 crianças participaram da segunda etapa, incluindo avaliação audiológica básica e comportamental do PAC.

**Resultados:**

A variável sexo influenciou a tarefa DD à orelha direita. A idade influenciou o resultado de cinco tarefas auditivas. A orelha direita teve melhor desempenho nas tarefas DD e OT-F. Na idade entre 6 e 7 anos houve correlação entre triagem e diagnóstico nas tarefas de FA, RT, DD, FF e OT-F. Na idade de 8 anos houve correlação nas tarefas DD e OT-F. Os critérios de passa/ falha variaram de acordo com a tarefa e determinantes biológicos.

**Conclusão:**

Houve correlação entre triagem e diagnóstico em um maior número de tarefas na faixa etária entre 6 e 7 anos. Os pontos de corte das tarefas auditivas devem ser analisados de acordo com a idade, sexo e/ou lado da orelha.

## INTRODUCTION

The integrity and proper functioning of the peripheral and the central auditory nervous systems (CANS) are fundamental for school success. Central auditory processing (CAP) is the perceptual processing of auditory information in the CANS and the neurobiological activity that underlies that processing and gives rise to electrophysiologic auditory potentials^(1)^. American Speech-Language-Hearing Association recommends the administration of a test battery with at least one test evaluating each mechanism/skill, including sound localization and lateralization; auditory discrimination; auditory pattern recognition; temporal aspects of audition, including temporal integration, temporal discrimination (e.g., temporal gap detection), temporal ordering, and temporal masking; auditory performance in competing acoustic signals (including dichotic listening); and auditory performance with degraded acoustic signals^(1)^. Researchers have documented that student with low performance in reading and/or writing skills commonly present changes in CAP auditory skills, with emphasis on the dichotic listening and temporal processing mechanisms^(2,3)^.

Hearing screening actions in the school environment that are directed at CAP, should be inserted in prevention and health care routines, with emphasis on students in the early years of the literacy process, since screening may favor the early identification of Children who need differential diagnostic assessment; contribute to actions that can increase the awareness of educators and parents regarding the importance of hearing skills^(4)^. An effective tool in the selection of Children who need to be referred for future diagnostic evaluation of CAP is needed. Early diagnosis allows for the assertive referral of Children who will benefit from auditory skills stimulation therapy, consequently minimizing losses from an educational point of view^(5-7)^.

From 1986 onwards, there was a growing effort by researchers to develop a CAP screening battery that included different hearing skills^(8)^. The *Screening Test for Auditory Processing Disorder* (SCAN) was the first battery, with later versions for adults (SCAN-A) and Children (SCAN-C)^(9,10)^, however, the battery did not include screening mechanisms for temporal auditory processing, permeating the search for a new comprehensive tool of auditory skills. In later years additional screening batteries were developed, such as the Multiple Auditory Processing Assessment (MAPA), which included the skills of binaural integration, binaural separation, auditory closure and temporal ordering^(11)^; the Screening Test for Auditory Processing (STAP) that contains subtest, accessing Speech Perception in Noise, Dichotic Consonant-Vowel, Gap Detection and Auditory Memory^(12)^ and a computer program called Feather Squadron that screens for five of the six hearing mechanisms recommended by the American Speech-Language-Hearing Association (ASHA), including sound localization, auditory patterns, temporal aspects of hearing, dichotic listening with competitive acoustic signal and auditory performance with degraded acoustic signal^(13)^.

Despite advances in audiology, there is still a gap and the need for effective procedures for CAP screening, as there is no consensus on the most efficient protocol to be used^(14)^. ASHA also recommends the use of checklists or questionnaires based on psychometric principles in screening actions, considering the complexity involved in the CAP^(1)^. The judgment of parents and/or teachers regarding the child's auditory behavior is extremely important in order to detect those at risk for changes and it should not be underestimated^(5,15,16)^.

In view of this scenario, a new hearing screening battery called AudBility was developed in Brazil. AudBility is a computational system that contains behavioral auditory tasks that access the six auditory mechanisms recommended by ASHA, in addition to psychometric questionnaires. The development of the tool, as well as the description of the initial version, was previously reported^(17)^. Based on this study, the authors recommended the creation of two modules, one with illustrative figures that do not require reading and writing skills to be applied to Children under 9 years old or who do not yet have full writing skills and another module for Children above 9 years old with tasks involving written words. The module aimed at students under 9 years of age was studied and the results recently published^(18)^. This article is linked to the aforementioned study, which aimed to analyze the sensitivity and specificity data analyzed according to the influence of the biological determinants, which are presented in this present study. Such data were still in the submission/peer review phase at the time of its publication. In the aforementioned study, the data related to the different cutoff points in each task in relation to the sensitivity and specificity of the battery were presented, considering those tasks in which it was possible to construct the ROC curve, with at least five children altered in the diagnostic tests, however, the point of greatest accuracy was not highlighted considering each task studied.

Thus, it is necessary to present the points of best accuracy of the battery for each task according to the variables studied, as well as the cutoff point for the tasks in which it was not possible to construct the ROC curve, to contribute to clinical practice. Thus, the objective of this study is to analyze the performance of students between 6 and 8 years of age in a hearing skills screening software program - AudBility, considering the influence of biological determinants and the correlation of auditory tasks with the corresponding tests of the behavioral assessment of central auditory processing (CAP), as well as to present the cutoff points of pass/failure for the battery.

## METHODS

### Type, study location and ethical aspects

This is a descriptive, analytical, prospective, cross-sectional study of diagnostic accuracy. The study was carried out in two stages, with Stage 1 referring to the Central Auditory Processing (CAP) screening at a Public-School setting and Stage 2 referring to the CAP diagnostic at the institution's audiology laboratory facility. The study was approved by the institution's Research Ethics Committee, under opinion No. 2.294.609. The student’s parents/legal guardians gave consent for their voluntary participation in the research by signing the Free and Informed Consent Form (ICF) and the Children also signed a Term of Consent.

### Stage 1 - central auditory processing screening in a school setting

#### Sample

The study was carried out with schoolchildren recruited voluntarily in a convenience sample from a public school. Two hundred and three invitation letters were sent to parents/legal guardians and 157 (77%) of them agreed to participate in the research.

For this study, only students who met the inclusion/exclusion criteria were included.

*Inclusion/exclusion criteria:* Age group between 6 and 8 years old, native speakers of Brazilian Portuguese, normal peripheral screening procedures, child without previous diagnostic of cognitive changes/syndromes or neurodevelopmental disorders.

*Additional Exclusion criteria:* Children who did not have adequate understanding during the screening battery.

Based on the defined criteria, the sample at this stage was composed by 96 students, 50 of whom were female and 46 were male. The participants’ average age was 7.47 +0.97 years old.

#### Procedures

Initially, teachers responded to a survey about each child, regarding their school performance, auditory behaviors, and relationships with their peers (Appendix A).

Hearing screening was conducted by the researcher, who is a signatory to this study, in a computer room provided by the school. Each child was screened individually. Firstly, an otoscopy (Hein otoscope) was performed and immittance measures (MT-10 Interacoustics equipment) were taken, including tympanometry and ipsilateral acoustic reflex research at 500Hz, 1KHz, 2KHz and 4KHz. Only children with type A tympanometric curves and a presence of acoustic reflexes continued to perform the AudBility tasks.

**Figure 1:**
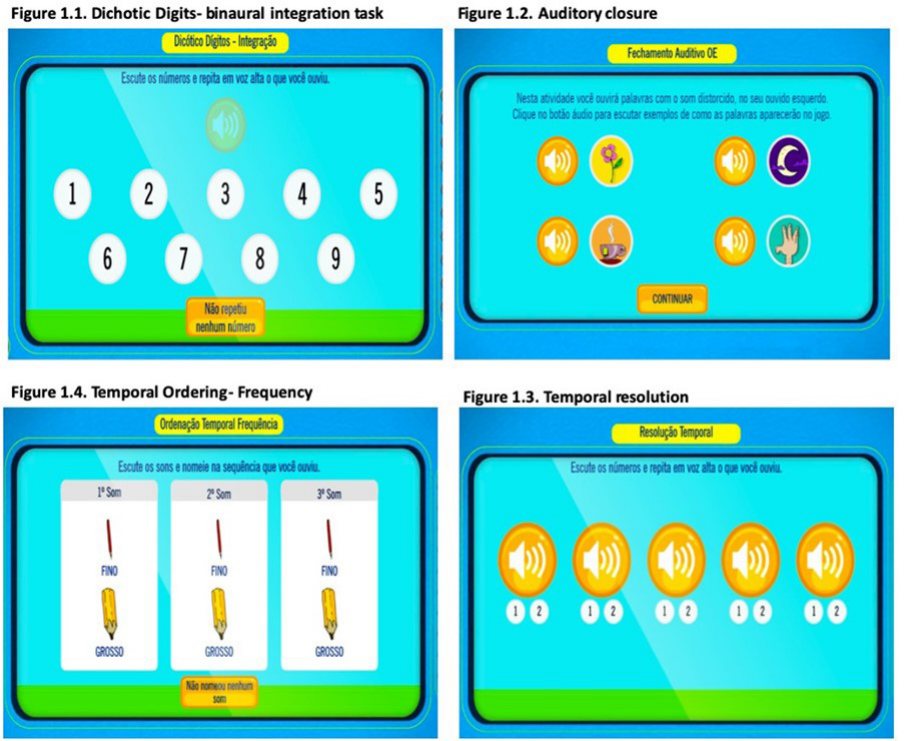
Examples of illustrations of auditory tasks

**Figure 2:**
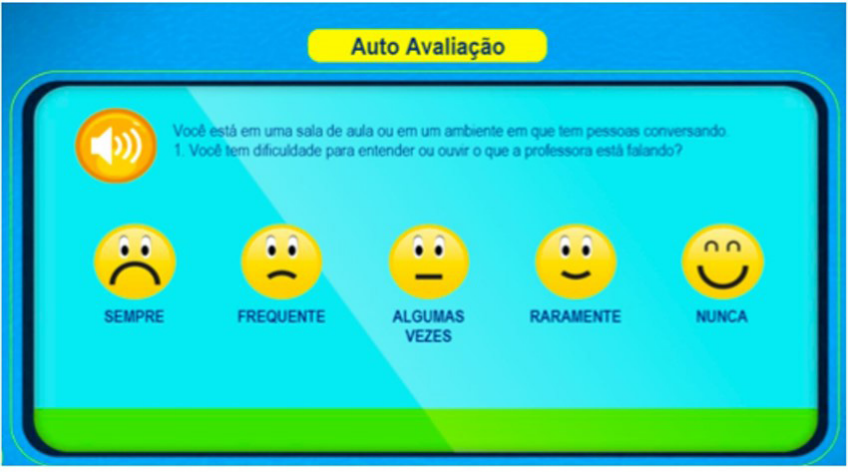
Example of question 1 of the questionnaire - additional questions in Appendix B

AudBility offers the possibility of computerized management of collected data, which enabled the creation of a database to store outcomes and allows for the visualization of the performance of each task in terms of percentage and/or number of correct answers right after the task or at the end of the screening^(17)^. For the application of AudBility, a desktop computer with online access was provided by the school. The computer's volume mixer was set at 50% and the child used a noise-canceling headset, Panasonic supra-headset model: RPHC720. Below are examples of the illustrations of the applied auditory tasks, in addition to the Auditory Processing Self-perception Questionnaire (QAPAC) (Figures 1 and 2).

**Caption:** 1.1 Dichotic Digits integration. Listen to the numbers and repeat out loud what you heard

1.2 Auditory Closure - left ear. In this activity, you will listen to words with missing parts in your left ear. Click the audio button to listen to the examples as they will appear in the game

1.3 Temporal resolution. Listen to the sounds and repeat out loud what you heard

1.4 Temporal Ordering Frequency. Listen to the sounds and repeat the sequence you Heard. Thick sound and Bold sound. Did not hear any sound

**Caption:** Self- assessment. Imagine you are in a classroom or in a room with people talking

1. Do you have a hard time understanding what the teacher is saying? Always, Frequently, Sometimes, Rarely, Never

The auditory tasks and the QAPAC answered by the student are described below:

1- **Sound Localization (SL):** 10 situations in which the child hears sounds that represent everyday activities, such as the sound of a mosquito flying, the tearing of paper, fingers snapping, steps on the stairs, among others. The child must choose the correct direction that the sound comes from, with respect to the location of the target stimulus (right, left, back / up or right and left).
2- **Auditory Figure-Ground (FG):** 10 sequences per ear in which the child hears a story and concomitantly a sentence referring to the figure. The child should ignore the story and point out to the picture corresponding to the target message. Five sentences are presented in a signal-to-noise ratio of -10dB, considering “noise”, with history and the signal being the target phrase of the test, and 5 sentences are presented in the signal-to-noise ratio -15dB. At the end, the percentage of correct answers by condition (in a total of five sentences each) and the percentage of correct answers in the test are presented.
3- **Dichotic Digits- Binaural Integration (DD):** 10 sequences in which the child hears 4 numbers concomitantly (two in the right ear and two of them in the left ear). The child should repeat and/or point to the four numbers heard as an answer, regardless of the listening order. The screen will always show options 1 to 9 so that the four digits heard are chosen. At the end, the percentage of correct answers, per ear (20 digits in each) and the percentage of correct answers are presented.
4- **Auditory Closure (AC):** 10 sequences per ear in which the child hears an acoustically distorted word and must recognize the word from among the figures presented. The options consist of four-word options and the “other word” option. The distortions were produced using the Gargle effect option.
5- **Temporal Resolution (TR):** Initially the child becomes familiar with the stimuli of the activity, which is a simple stimulus (whistle) with intervals between them - the gaps- which have variations of 20ms, 15ms, 10ms, 6ms, 4ms and 0ms. In each presentation, the child hears a sequence of six sounds and is instructed to count how many he/she can perceive/hear. At the end, the child says the answer. The number of gaps can vary from 1 to 2 double stimuli in each sequence. At the end, the total percentage of correct answers in 10 presentations is displayed. Throughout the 10 tracks the number of gaps is presented at random.
6- **Temporal Ordering Duration (TO-D):** The child listens to the stimuli of the activity, with a pure tone of 800Hz lasting 400ms (LONG) and another lasting 200ms (called CURTO-C). The activity consists of 10 sequences of three combinations between these pure tones with a silence time between them of 350ms, such as LLC, CCL, LCL, CLC, CLL and LCC. Half of the strings are presented in the right ear and half in the left ear. The child must hear and name the correct sequence.
7- **Temporal Ordering Frequency (TO-F):** Initially the child was familiarized with the activity stimuli, being a severe stimulus (called GROSSO-G) of 700Hz and an acute stimulus (called FINO-F) of 1500 Hz. The activity consists of 10 sequences of three combinations between these pure tones lasting 350ms, such as GGF, FFG, FGF, GFG, GFF and FGG. Half of the strings are presented in the right ear and half in the left ear. The child must hear and name the correct sequence.
8- **Auditory Processing Self-perception Questionnaire (QAPAC)** based on “Scale of Auditory Behaviors” on its translated version for European Portuguese^(19)^. The questionnaire is inserted in the software's online platform, contains 12 daily situations followed by questions on a Likert-type scale (12 to 90 points) with five closed response alternatives. The researcher reads the questions and selects the answer given by the child individually on the screen. The child responds with respect to how often the event or difficulty occurs (Frequent- Often- Sometimes- Seldom- Never) according to the attached template (Appendix B).

All students that took part in the first stage were invited to attend the institution's audiology laboratory for the CAP diagnostic. The pure tone screening research cannot be performed immediately, therefore, the children were referred within 72 hours to perform the procedure, included in stage 2. A similar method was applied in a previous study^(13)^.

### Stage 2 - central auditory processing diagnostic

The sample of the second stage consisted of 66 students. At this stage, to confirm the good academic performance the data was obtained from the children’s teacher and their school records and later confirmed in the interview/case history with the parents. Thus, the sample of the second stage consisted of 66 students, 37 of whom were female and 29 were male, being 25 children aged 6 years, 14 children aged 7 years and 27 children aged 8 years. Therefore, in stage 2, all children underwent basic audiologic assessment, consisting of otoscopy, pure tonal audiometry, logoaudiometry and immittance measures (tympanometry and ipsilateral and contralateral acoustic reflexes) to confirm the integrity of the peripheral auditory system obtained with the portable device in the screening stage. The normality criteria for basic audiological evaluation included tonal auditory thresholds up to 15dB in all frequencies (250 to 8KHz), speech reception threshold compatible with the tritonal mean of 500, 1000, and 2000Hz, speech recognition index above 88%, as well as a tympanometric curve with maximum compliance peak around the atmospheric pressure of 0 daPa and equivalent volume from 0.3 to 1.3 ml (type A)^(20)^. The evaluations were performed in an acoustic booth, using a duly calibrated AC40- Interacoustics audiometer and TDH 49 headphones and a Dell brand notebook.

The CAP behavioral diagnostic battery protocol was performed after confirming peripheral hearing evaluation normality. The CAP diagnostic evaluation battery protocol included auditory tests standardized for Brazilian Portuguese, equivalent to each AudBility task. Therefore, the SL task was correlated with the Masking Level Difference (MLD)^(21)^; the TR task with the Random Interval Detection Test (RGDT)^(22)^, the TO-F task with the Frequency Pattern Test (FPT)^(23)^; the DD task with the Dichotic Digit test (DD)^(24)^, the AC task with the Speech in Noise (SN) test^(24)^ and the FG task with the Identification test Pediatric sentences with Ipsilateral Competitive Message (PSI) in the signal-to-noise ratio -15dB^(24)^. In the diagnostic stage, to reduce the time spent during assessment, the temporal ordering of duration ability was not tested, therefore, the temporal ordering of duration ability screening task was not correlated with the diagnostic test.

The established criterion for the diagnosis of central auditory processing disorder (CAPD) was below-average performance for at least one ear in at least two different behavioral auditory processing tests, considering standardization for Brazilian tests for each age group^(24)^. Only Children with normal CAP (N=55), being 29 of them in the 6- and 7-year-old age group and 26 in the 8-year-old age group, were considered for the establishment of the cutoff point for the screening tasks based on the minus one standard deviation (SD) average when it was not possible to obtain it through the construction of the ROC curve in the sensitivity and specificity study^(18)^.

### Analysis

The analyzes of the cut-off points presented here were primarily based on the method performed in the previous study^(18)^, considering the best balance between sensitivity and specificity, that is, the point at which J reached its highest value, by calculating the Youden’s J index^(25)^. Since the construction of the ROC curve requires a significant number of individuals in the two sets that need to be differentiated (in this case, Normal and Altered), tests with less than five individuals in the Altered or Normal group could not have their values calculated. Therefore, in order to achieve the objectives of the present study, we chose to obtain the cut-off point of these screening tasks and the correlation analysis based on the performance of children with good school performance and normality in the behavioral assessment of the PAC. Descriptive statistics were calculated by means of descriptive analysis of mean, standard deviation, median, minimum and maximum referring to the total final score of the questionnaire and the percentage of correct answers for each auditory task. The value of statistical significance adopted was 5% (p≤ 0.05). The SPSS Statistics software, version 25.0 (IBM Corp., Armonk, NY, USA) was used. To calculate 95% confidence intervals, the corrected and accelerated bias method was used based on 2000 bootstrap samples^(26)^. For the correlation analysis, the correlation coefficient and the p value were calculated using Pearson or Spearman correlation tests.

## RESULTS

Tables 1 and 2 show the analysis of the influence of the gender and age variables, respectively. In Table 1, the Student's t-test for independent samples was used. The influence of the gender variable on AudBility was observed only in the DD task on the right ear. There was no statistically significant difference between the responses obtained in the self-perception questionnaire for males and females (Table 1).

**Table 1:** Descriptive values and comparative analysis of the performance of male and female children in the AudBility tasks

Tasks	Gender	Average	SD	Median	Min.	Max.	p
SL (%)	Female	84.40	13.43	90.00	40.00	100	0.467
	Male	86.30	11.99	90.00	60.00	100	
AC - RE (%)	Female	92.40	10.41	100	50.00	100	0.846
	Male	92.83	11.09	100	60.00	100	
AC -LE (%)	Female	90.60	16.71	100	20.00	100	0.538
	Male	92.39	10.79	100	50.00	100	
TR - Threshold (ms)	Female	4.96	3.34	4.00	4.00	20.00	0.654
	Male	4.67	2.84	4.00	4.00	20.00	
DD -RE (%)	Female	89.00	9.74	90.00	65.00	100	0.017*
	Male	93.16	6.78	95.00	75.00	100	
DD -LE (%)	Female	85.8	13.52	90.00	45.00	100	0.695
	Male	84.78	11.68	90.00	50.00	100	
FG - RE (%)	Female	86.60	15.99	90.00	10.00	100	0.603
	Male	88.04	10.25	90.00	50.00	100	
FG - LE (%)	Female	85.00	15.81	90.00	30.00	100	0.318
	Male	87.83	11.14	90.00	40.00	100	
TO-F - RE (%)	Female	75.60	26.89	80.00	0.00	100	0.122
	Male	83.48	22.03	90.00	0.00	100	
TO-F - LE (%)	Female	70.40	25.95	80.00	0.00	100	0.152
	Male	77.83	24.30	80.00	0.00	100	
TO-D - RE (%)	Female	32.80	27.92	30.00	0.00	100	0.530
	Male	36.52	29.90	20.00	0.00	100	
TO-D - LE (%)	Female	28.40	27.43	20.00	0.00	100	0.434
	Male	33.04	30.47	20.00	0.00	100	
QAPAC	Female	47.04	7.53	50.00	27.00	56.00	0.196
	Male	45.13	6.78	45.00	31.00	59.00	

Student's t-test for independent samples

* Statistically significant value at the 5% level (p ≤ 0.05)

**Caption:** SD = Standard deviation; Min. = Minimum; Max. = Maximum; RE= Right ear; LE= Left ear

**Table 2:** Descriptive values and comparative analysis of the performance between age groups in the AudBility tasks

Tasks	Age	Average	SD	Median	Min.	Max.	p
SL (%)	6	83.25	13.47	85.00	40.00	100	0.087
	7	83.16	10.57	80.00	70.00	100	
	8	88.65	12.51	90.00	60.00	100	
AC- RE (%)	6	90.50	10.85	90.00	50.00	100	**< 0.001**
	7	88.42	13.02	90.00	60.00	100	
	8	97.03	7.40	100	70.00	100	
AC -LE (%)	6	85.75	18.80	90.00	20.00	100	**< 0.001**
	7	91.58	8.34	90.00	80.00	100	
	8	97.57	5.97	100	70.00	100	
TR - Threshold (ms)	6	5.83	4.62	4.00	4.00	20.00	0.127
	7	4.11	0.46	4.00	4.00	6.00	
	8	4.11	0.46	4.00	4.00	6.00	
DD - RE (%)	6	87.00	9.40	87.50	65.00	100	0.001*
	7	93.16	6.28	95.00	75.00	100	
	8	94.18	7.22	95.00	70.00	100	
DD - LE (%)	6	78.62	13.92	85.00	45.00	95.00	**< 0.001**
	7	86.84	10.96	90.00	50.00	95.00	
	8	91.76	7.56	95.00	65.00	100	
FG - RE (%)	6	83.25	16.07	90.00	10.00	100	0.020*
	7	88.95	11.97	90.00	50.00	100	
	8	90.81	9.83	90.00	60.00	100	
FG - LE (%)	6	82.75	16.64	90.00	30.00	100	0.058
	7	89.47	13.11	90.00	40.00	100	
	8	88.65	9.48	90.00	60.00	100	
TO-F - RE (%)	6	70.00	30.04	80.00	0.00	100	0.013*
	7	82.11	19.88	80.00	40.00	100	
	8	88.11	16.64	100	40.00	100	
TO-F - LE (%)	6	61.00	26.00	60.00	0.00	100	**< 0.001**
	7	72.63	21.30	80.00	20.00	100	
	8	88.65	17.98	100	40.00	100	
TO-D - RE (%)	6	18.00	17.42	20.00	0.00	60.00	**< 0.001**
	7	31.58	29.30	20.00	0.00	100	
	8	54.05	27.02	60.00	0.00	100	
TO-D - LE (%)	6	17.00	20.53	20.00	0.00	80.00	**< 0.001**
	7	34.74	32.55	20.00	0.00	100	
	8	43.24	28.87	40.00	0.00	100	
QAPAC	6	45.68	7.26	46.00	30.00	56.00	0.828
	7	46.89	7.91	49.00	33.00	59.00	
	8	46.22	6.94	47.00	27.00	56.00	

Kruskal-Wallis Test

* Statistically significant value at the 5% level (p ≤ 0.05)

**Caption:** SD = Standard deviation; Min. = Minimum; Max. = Maximum; RE= Right ear; LE= Left ear

The influence of the age variable on AudBility showed a statistically significant difference in five auditory tasks using the Kruskal-Wallis Test. There was no statistically significant difference between the responses obtained in the self-perception questionnaire between the age groups (Table 2).

A post hoc analysis was performed with the Mann-Whitney U test (Table 3) to identify in which age pairs the differences were statistically significant.

**Table 3:** Comparative analysis between age pairs in AudBility tasks

Tasks	Post-hoc analysis - Pair comparison					
	6 x 7		6 x 8		7 x 8	
	p	E.S.	p	E.S.	p	E.S.
AC - RE (%)	> 0.999	0.051	0.002*	0.393	0.005*	0.423
AC -LE (%)	> 0.999	0.090	< 0.001*	0.480	0.019*	0.364
DD - RE (%)	0.059	0.304	< 0.001*	0.431	> 0.999	0.101
DD - LE (%)	0.084	0.286	< 0.001*	0.548	0.261	0.229
FG - RE (%)	0.221	0.233	0.022*	0.305	> 0.999	0.053
TO-F - RE (%)	0.551	0.173	0.010*	0.334	0.869	0.142
TO-F - LE (%)	0.457	0.186	< 0.001*	0.587	0.018*	0.368
TO-D - RE (%)	0.339	0.206	< 0.001*	0.617	0.015*	0.376
TO-D - LE (%)	0.096	0.279	< 0.001*	0.472	0.653	0.165

Mann-Whitney U test with Bonferroni correction for multiple comparisons

* Statistically significant value at the 5% level (p ≤ 0.05)

**Caption:** E.S. = Effect size; RE= Right ear; LE= Left ear

The influence of the ear side on the CAP screening battery showed a statistically significant difference with the Student's t-test for paired samples, with better performance of the right ear in the DD and TO-F tasks (Table 4).

**Table 4:** Descriptive values and comparative analysis between the sides of the ears in relation to performance in AudBility

Tasks	Ear	Average	SD	Median	Min.	Max.	p
AC (%)	Right	92.60	10.69	100	50.00	100	0.364
	Left	91.46	14.14	100	20.00	100	
DD (%)	Right	90.98	8.66	95.00	65.00	100	< 0.001*
	Left	85.32	12.62	90.00	45.00	100	
FG (%)	Right	87.29	13.49	90.00	10.00	100	0.424
	Left	86.35	13.77	90.00	30.00	100	
TO-F (%)	Right	79.38	24.87	80.00	0.00	100	0.019*
	Left	73.96	25.32	80.00	0.00	100	
TO-D (%)	Right	34.58	28.80	20.00	0.00	100	0.084
	Left	30.63	28.87	20.00	0.00	100	

Student's t-test for paired samples

* Statistically significant value at the 5% level (p ≤ 0.05)

**Caption:** SD = Standard deviation; Min. = Minimum; Max. = Maximum

66 students participated in the diagnostic stage and the adherence was 68%. The correlation coefficient (coef.) between the performance in the screening tests and the respective CAP diagnostic tests according to the age group was obtained. In the age group between 6 and 7 years old, there was a statistically significant (p ≤ 0,05) and positive correlation (directly proportional), indicating that the increase in one of the variables was associated with the increase in the other variable in the respective screening and diagnostic tests: Auditory Closure (AC) screening and SN diagnostic test (coef. 0.387), Temporal Resolution (TR) screening and RGDT diagnostic test (coef. 0.409), Dichotic Digits- Binaural Integration (DD) screening and DD diagnostic test in the left ear in females (coef. 0.428), Dichotic Digits- Binaural Integration (DD) screening and DD diagnostic test in the right ear in males (coef. 0.692), Auditory Figure-Ground (FG) screening and PSI diagnostic test (coef. 0.245), Temporal Ordering Frequency (TO-F) screening and FPT diagnostic test in the right ear (coef. 0.715) and Temporal Ordering Frequency (TO-F) screening and FPT diagnostic test in the left ear (coef. 0.614).

At 8 years of age, there was a statistically significant and positive correlation in the screening for DD and the DD diagnostic test in the left ear in females (coef. 0.726) and in TO-F screening and the FPT diagnostic test in the right ear (coef. 0.437).

After completing the application of the CAP behavioral assessment battery, normality was found in 55 Children (83.33%). Only these Children were considered for the establishment of the cutoff point in the screening tasks based on the -1 SD average in the tasks of sound localization, dichotic digits, auditory closure, and temporal ordering duration (Table 5).

**Table 5:** Cuttof point performance for schoolchildren aged 6 to 8 years old in the AudBility tasks

Tasks (AudBility)	Cutoff point-based roc curve^(18)^		Cutoff point - Average - 1 SD DP		Variation of pass/fail criteria
	6 to 7 years old	8 years old	6 to 7 years old	8 years old	
Sonorous localization (%)	-	-	71.78	81.22	2-3 errors
Auditory closure (%)	85	-	-	92.23	1-2 errors
Temporal Resolution - Threshold (ms)	5 ms	5 ms	-	-	threshold 5 ms
Figure-ground (%)	75	85	-	-	2-3 errors
Digits - Binaural Integration - RE ♀ (%)	77.5	-	-	89.80	6 errors- female
Digits - Binaural Integration - RE ♂ (%)	-	80	85.71	-	4 errors -male
Digits - Binaural Integration - LE ♀ (%)	72.5	87.5	-	-	
Digits - Binaural Integration - LE ♂ (%)	87.5	-	-	86.07	
Temporal Ordering Frequency - RE (%)	70	50	-	-	1- 2 errors
Temporal Ordering Frequency - LE (%)	70	90	-	-	
Temporal Ordering Duration (%)	-	-	2.46	28.77	

**Caption:** SD = Standard deviation; RE= Right ear; LE= Left ear

## DISCUSSION

The screening battery called “AudBility” is a new CAP screening tool in the Brazilian context, and it has demonstrated feasibility for application on schoolchildren starting at six years of age in a school setting^18^. The average battery application duration was 30 minutes, a duration commonly found in previous screening work that proposed to comprehensively screen CAP skills^(11,13)^.

The gender variable demonstrated not to influence the performance of most screening tasks, however, in the DD to the right ear task, males had a better performance than females. This finding was not observed in a previous hearing skills screening study conducted with English-speaking schoolchildren, aged 5 to 7 years old^(13)^. However, male’s greater sensitivity in central hearing skills have already been reported in a study that applied the standard frequency test^(27)^ and in a study that applied the frequency modulation test^(28)^, but the understanding of this sensitivity is not yet clear and further studies must be carried out for confirmation.

The age variable has been shown to influence the performance of most auditory tasks that involve later maturation of the central auditory nervous system (CANS). The 8-year-olds performed significantly better compared to the 6-year-olds. The age of 7 proved to be a transition age. The effect of age on the performance of CAP screening and diagnostic tasks is expected and compatible with CANS maturational development^(2,13,29)^. Therefore, it is important to highlight that although children aged six years did not present a statistically significant difference from children aged seven years, these data should be interpreted only as a screening criterion, which aims at the early identification of at-risk children, allowing early stimulation until maturation for diagnosis at seven years of age.

The ear side variable had a significant effect on the performance of the DD and TO-F tasks, with better performance on the right ear. A recent study also found better results for the right ear compared to the left ear in the DD test in a group of Children with normal results in the CAP battery^(30)^. A better performance of the right ear is expected in auditory mechanisms that require the crossing of auditory information via the corpus callosum in this age group, compatible with the screening findings. According to Kimura's theory, the contralateral neural pathways contain more fibers than the ipsilateral pathways, so the verbal stimuli presented in the right ear have direct and rapid access to the left hemisphere, while the stimuli presented to the left ear reach the right hemisphere first, and only after transmission via the corpus callosum do they reach the left hemisphere^(31)^. In temporal ordering tasks, which require naming by the child, as conducted in this study, it requires the processing of both hemispheres, that is, it first requires the processing of the acoustic contour in the right hemisphere and the transfer via the corpus callosum to the hemisphere left for linguistic labeling^(32)^.

The Children's performance in the self-perception questionnaire was not influenced by gender or age, suggesting that it can be applied starting at the age of six. The average of the final score in the Children's questionnaire in the present study (46.13±7.21) was similar to the values obtained in recent studies published in the literature, which used the same self-perception questionnaire with students with good school performance, with a mean age of 9.58 and 9.6 years, and found an average score of 46.7±6.44 and 44.75±6.3, respectively^(15,17)^.

The correlation between the AudBility auditory tasks with the respective tests of the diagnostic battery showed that in the age group between 6 and 7 years old there was a statistically significant and positive correlation, suggesting that the increase of one of the variables was associated with the increase of the other variable, in the AC, TR, DD, FG and TO-F tasks. These findings suggest an important contribution by AudBility to the early referral of Children early in the literacy process.

In the 8-year-olds age group, there were statistically significant and positive correlations in two tasks only, DD and TO-F. However, despite the lower number of tasks having a correlation with the diagnosis, it is noteworthy that these two tests are important and commonly altered in Children diagnosed with central auditory processing disorder (CAPD), having already been reported that 82% of Children diagnosed with CAPD scored below normal limits in the frequency pattern test and 54% in the dichotic digit test^(30)^. Another study found that the frequency pattern test is the test in which Children with CAPD comorbidities, language and reading difficulties have the worst results, according to the authors, this is due to the complexity of the test^(2)^.

It is important to note that since 1990, researchers have recommended that a CAP screening program included at least one dichotic listening test due to its short application time, sensitivity to detect central injuries and to be relatively resistant to mild hearing loss; a low redundancy monoaural test and a temporal ordering test^(4)^, being exactly two of the three suggested tests in which correlations between the screening tasks and the diagnostic tests were observed in 8-year-old Children.

In the present study, the temporal ordering of duration task proved to be difficult for Children in the 6 to 8-year-old age group, because in all ages they had Children who were unable to score in the task, causing a high SD and a low cutoff point. Therefore, the temporal ordering of duration diagnostic test was not applied in the second stage of the study to perform the correlation analysis due to the difficulty presented by the Children in the screening. Therefore, it is possible to suggest that the TO-D task should not be part of a screening protocol in this age group, being more appropriate to only maintain the TO-F task.

The cutoff points established for Children with normal CAP battery demonstrated that age should be considered when analyzing the child's performance in hearing screening, as well as the performance of the ear in the DD and TO-F tests because the Children are still undergoing maturation. CAP screening should cover schoolchildren with and without academic complaints because 16.67% of Children with no previous complaints and considered to have good school performance were diagnosed with CAPD, and it may interfere in social and/or communication situations in the future, if the altered skills are not stimulated properly.

The results of this study are important because a recent study with AudBility showed effectiveness in sensitivity and specificity values, therefore, have been referred to in the literature as relevant battery for screening schoolchildren^(18)^. In addition, it is necessary to know the normality criteria for greater coverage of the use of the software program, both in clinical and school screening settings.

The study has limitations in relation the evaluation of school performance considered only the perceptions of parents and teachers as well as each participant’s case history, without the application of formal tests to assess language and cognitive aspects. However, the normality of CAP in 83% of the Children who attended the diagnostic battery contributed to the establishment of the cut-off point for AudBility.

## CONCLUSION

Based on the data from the present sample, we suggest that the cutoff points for auditory tasks should be analyzed according to age, gender, and side of the ear. There was a correlation between screening and diagnosis in a greater number of tasks in the 6 to 7-year-old age group when compared to the 8-year-old age group.

## Appendix A Teacher survey

Date: ____ / ____ / ____

Name of the student: _________________________________________ Sex: () M () F

Age: ________ Schooling: ________________ Class (class): __________________

Name of the responsible teacher: _________________________________________

Dear Teacher,

Please answer the questions below carefully. In case of doubt, contact us for any clarification.

Yes No

Is the student participative in the classroom? () ()

Does the student have good school performance? () ()

Is the student attentive and focused on activities? () ()

Does the student favor any subject? () ()

If YES, which one? ____________________________________

Does the student behave well at school? () ()

Does the student interact with other children / adults? () ()

Do you notice signs of respiratory and / or hearing disorders? () ()

Does the student have difficulty / exchange of phonemes in speech? () ()

Does the student have difficulty / exchange of phonemes in writing? () ()

Does the student understand the texts well? () ()

Comments:_____________________________________________________________________________________________________________________________________________________________________________________________________________________________________________________________________________________________________

## Appendix B Questionnaire auditory processing self perception (QAPAC)

Child name: _______________________________ School year: ____________

Please answer based on your experience. If you are not sure, refer to comments you have heard about yourself. Each question should be evaluated based on how often the situation occurs or not.

1. You are in a classroom or environment where people are talking.

Do you have difficulty hearing or understanding what the teacher is saying? FREQUENT OFTEN SOMETIMES SELDOM NEVER

2. The teacher or a person is speaking with you very fast.

Do you have difficulty understanding what the teacher says?

FREQUENT OFTEN SOMETIMES SELDOM NEVER

3. The teacher or a person is giving you oral instructions (explanations).

Do you have difficulty following the oral instructions?

FREQUENT OFTEN SOMETIMES SELDOM NEVER

4. The teacher or a person is talking to you in a quiet environment.

Do you have difficulty hearing and understanding clearly the words without exchanging any letters?

FREQUENT OFTEN SOMETIMES SELDOM NEVER

5. When the teacher or a friend is talking to you.

Do you feel that sometimes you hear well and sometimes not?

FREQUENT OFTEN SOMETIMES SELDOM NEVER

6. You are in the classroom or school yard and someone calls out your name.

Do you have difficulty noticing where the sound comes from?

FREQUENT OFTEN SOMETIMES SELDOM NEVER

7. The teacher or a person is talking to you.

Do you ask them to repeat what they said?

FREQUENT OFTEN SOMETIMES SELDOM NEVER

8. You are in the classroom.

Do you get easily distracted?

FREQUENT OFTEN SOMETIMES SELDOM NEVER

9. Last year at school.

Did you have learning difficulties?

FREQUENT OFTEN SOMETIMES SELDOM NEVER

10. You are doing an assignment.

Do you have difficulty focusing?

FREQUENT OFTEN SOMETIMES SELDOM NEVER

11. When you are in the classroom or at home.

Do people say you daydream or are inattentive?

FREQUENT OFTEN SOMETIMES SELDOM NEVER

12. When you are at school or at home.

Are you disorganized?

FREQUENT OFTEN SOMETIMES SELDOM NEVER
